# Use of environmental DNA (eDNA) in streams to detect feral swine (*Sus scrofa)*

**DOI:** 10.7717/peerj.8287

**Published:** 2020-01-02

**Authors:** Amberly N. Hauger, Karmen M. Hollis-Etter, Dwayne R. Etter, Gary J. Roloff, Andrew R. Mahon

**Affiliations:** 1 Biology Department, University of Michigan—Flint, Flint, MI, United States of America; 2 Wildlife Division, Michigan Department of Natural Resources, East Lansing, MI, United States of America; 3 Department of Fisheries and Wildlife, Michigan State University, East Lansing, MI, United States of America; 4 Department of Biology, Central Michigan University, Mount Pleasant, MI, United States of America

**Keywords:** Feral swine, *Sus scrofa*, Environmental DNA, Streams, Water samples, Terrestrial mammal, Invasive species, eDNA, Water temperature

## Abstract

Invasive feral swine can damage ecosystems, disrupt plant and animal populations, and transmit diseases. Monitoring of feral swine populations requires expensive and labor-intensive techniques such as aerial surveys, field surveys for sign, trail cameras, and verifying landowner reports. Environmental DNA (eDNA) provides an alternative method for locating feral swine. To aid in detection of this harmful invasive species, a novel assay was developed incorporating molecular methods. From August 2017 to April 2018, water samples and stream data were collected along 400 m transects in two different stream types where swine DNA was artificially introduced to investigate potential factors affecting detection. A generalized linear model (family binomial) was used to characterize environmental conditions affecting swine DNA detection; detection was the dependent variable and stream measurements included stream type, distance downstream, water temperature, velocity, turbidity, discharge, and pH as independent variables. Parameters from the generalized linear model were deemed significant if 95% confidence intervals did not overlap 0. Detection probability for swine DNA negatively related to water temperature (*β* =  − 0.21, 95% CI [−0.35 to −0.09]), with the highest detection probability (0.80) at 0 °C and lowest detection probability (0.05) at 17.9 °C water temperature. Results indicate that sampling for swine eDNA in free-flowing stream systems should occur at lower water temperatures to maximize detection probability. This study provides a foundation for further development of field and sampling techniques for utilizing eDNA as a viable alternative to monitoring a terrestrial invasive species in northern regions of the United States.

## Introduction

Invasive feral swine (*Sus scrofa*) harm plant and animal populations, disrupt natural ecosystems, and transmit diseases to livestock, pets, and humans (Hunter, 2012). Feral swine are relatively new to northern latitudes of North America with scattered distribution and low localized abundance (Etter, Nichols & Hollis-Etter, in press); however, expansion of feral swine has potential to cause detrimental ecological and economic impacts (Snow, Jarzyna & VerCauteren, 2017). Considering that feral swine are efficient invaders, the remoteness of habitats potentially occupied, and relatively low human densities in the northern United States and Canada, feral swine could rapidly expand and establish populations. Standard techniques for detecting feral swine rely on reports from the public and through field investigations including aerial surveys, trail camera monitoring, baiting, radio-telemetry, and intensive field searches for sign (i.e., tracks, feces, wallows, and/or rooting; Etter, Nichols & Hollis-Etter, in press). These methods are labor intensive, costly, and do not consistently produce reliable information about the status of feral swine at low densities.

In 2014, Michigan researchers captured feral swine to study their movements and assess ecological damage (Gray, 2019). Researchers discovered it was difficult to locate individuals and over three years of intensive effort, only 18 animals were successfully captured; partially because densities were lower than anticipated. Satellite telemetry data collected from collared feral swine documented large-scale and frequent movements, large home ranges, and extensive use of wetlands, including animals frequently crossing open water (Gray, 2019). Most individuals occupied expansive woodland and wetland cover types that were difficult to access even with all terrain vehicles (ATV). To prevent establishment and potential spread of this invasive species, management agencies need reliable techniques for detecting low density populations. Additional research is needed on techniques to increase reliability and efficiency of detecting feral swine at low densities in northern habitats (Etter, Nichols & Hollis-Etter, in press).

Recent advancements in molecular techniques offer alternatives for documenting animal presence by utilizing a DNA signature in an environmental sample. Aquatic organisms, such as fish and amphibians, are presently the focus of environmental DNA (eDNA) development and detection techniques in controlled and field experiments (Nevers et al., 2018; Takahashi et al., 2018; Tillotson et al., 2018). The ability to detect and quantify animal DNA from freshwater is determined by the relationship between DNA excretion and degradation (Lindahl, 1993), coupled with the ability to acquire that DNA through field sampling. One study using a controlled microcosm experiment successfully used eDNA to detect six threatened freshwater species (amphibians, fish, mammals, insects, crustaceans), and then demonstrated the technique in ponds (Thomsen et al., 2012). Species detected included a semi-aquatic mammal, Eurasian otter (*Lutra lutra*; Thomsen et al., 2012). Williams, Huyvaert & Piaggio (2016) developed laboratory techniques to successfully detect swine eDNA from pig wallows in a controlled experiment conducted in a livestock facility. However, this technique has not been replicated in field conditions with variable water flow, chemistry, substrates, and environmental conditions. Using eDNA as a monitoring tool for feral swine may allow wildlife managers to detect animals at low density before populations become established, or to locate small groups of individuals across expansive landscapes to focus eradication efforts.

This research utilized eDNA technology to parameterize detection of swine DNA in free-flowing aquatic systems. Study objectives included: (1) determine whether swine DNA could be detected in free-flowing streams; (2) parameterize detection probability of swine DNA in natural stream systems by stream variables; and (3) recommend sampling techniques for future feral swine eDNA surveillance.

## Methods and Materials

### Study sites

**Figure 1:**
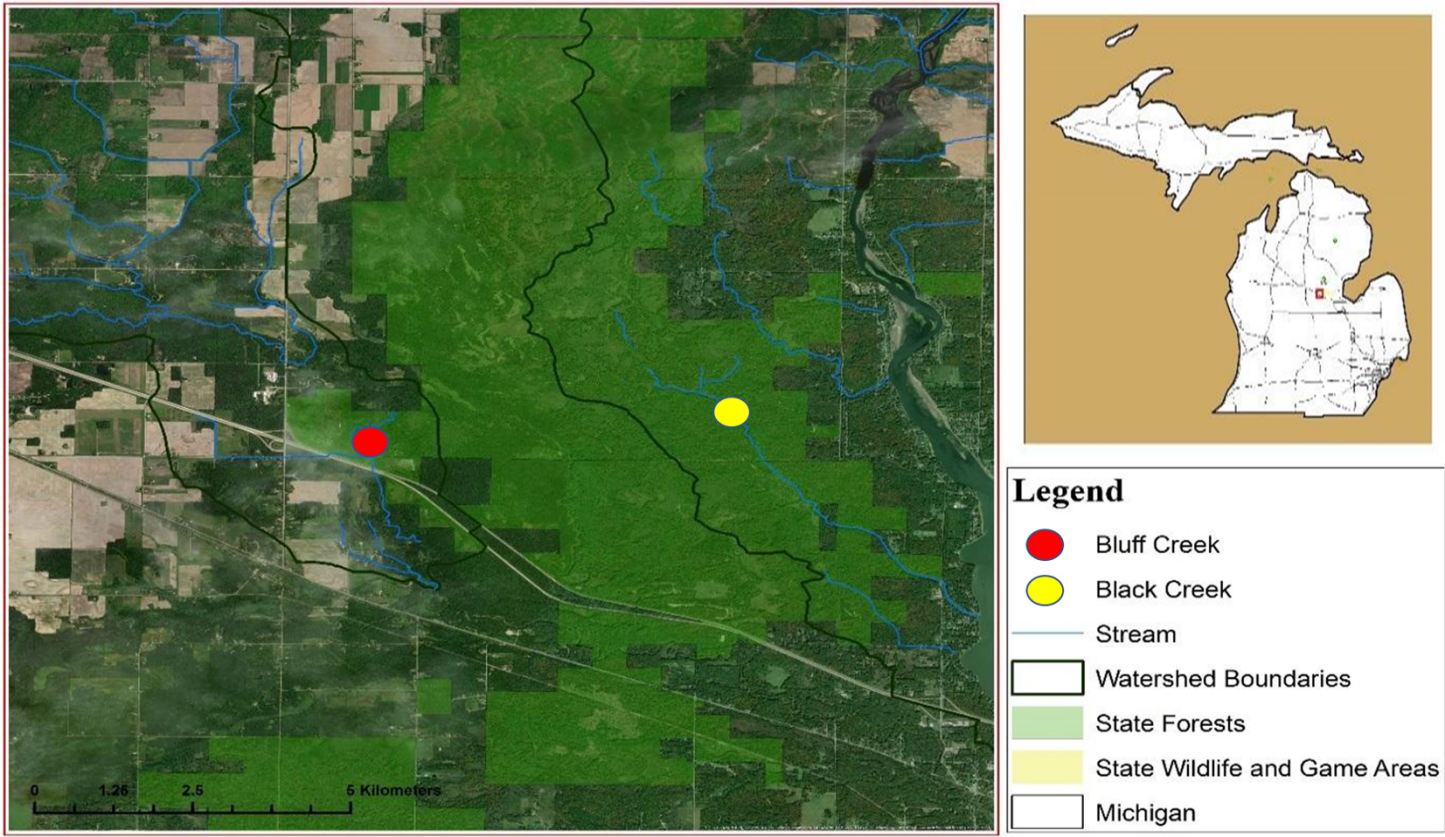
Location of watersheds containing Bluff and Black Creeks, Michigan, USA. Field sampling for swine DNA was conducted from August 2017 to April 2018. Data source: Esri DigitalGlobe.

This study was conducted in two free-flowing streams from August 2017 to April 2018. Bluff Creek is located near Sanford, Michigan (16N 01W Section 34, 43.72897 N, −84.47174 W) and Black Creek near Alamando, Michigan (16N 01W Section 31, 43.73348 N, −84.42216 W), both in Edenville Township, Midland County (Fig. 1). The Michigan Department of Natural Resources (MDNR) issued a state land use permit for access (#73-2017-013). Cover types included lowland shrubs, marshes, wetlands, beaver (*Castor canadensis*) ponds, and swamp hardwoods interspersed with upland oak (*Quercus* spp.) and aspen (*Populus* spp.) stands. Bluff Creek is characterized as uniform and free flowing with little streambank vegetation undergrowth, a full over story tree canopy, and narrow floodplain, with steep sloping banks that parallel the stream. Black Creek has a wide floodplain with multiple water channels when stream flow is high, and thick understory brush along the streambank.

### Monitoring for feral swine

Because the study design required artificially introducing swine DNA into streams at specific locations and times, inferring absence of feral swine within the study area was essential. Previous research in Michigan monitored the study area since 2013, with no evidence of feral swine detected (D Etter, pers. comm., 2017). The MDNR logs all reports of feral swine in the state to Township, Range and Section, and no reports were recorded for feral swine in Edenville Township from 2016 to 2018 (D Etter, pers. comm., 2019). Additionally, the Michigan Pork Producers had no records of any commercial swine operations within the immediate vicinity of the study sites (M Kelpinski, pers. comm., 2019). From November 2016 to April 2018, one week prior to DNA insertion and water sampling, field surveys were conducted along each stream for feral swine sign (tracks, scat, wallows, rooting; Mayer, 2009). Additionally, trail cameras (Bushnell® Trophy Cam HD Aggressor 14MP Trail Camera; Bushnell Outdoor Products, Overland Park, Kansas) were used to monitor wildlife trails and beaver dams along Bluff and Black Creeks. Ten cameras spaced 400 m apart were set over a total distance of 4000 m on each creek. Five cameras were placed upstream and five downstream from the DNA source point. Ten additional cameras were set in the Mud Creek watershed, located between Bluff and Black Creeks, for additional surveillance. Trail cameras were checked once every two weeks for the entirety of the study and reviewed for photos of feral swine.

### Field sampling methods and materials

**Figure 2:**
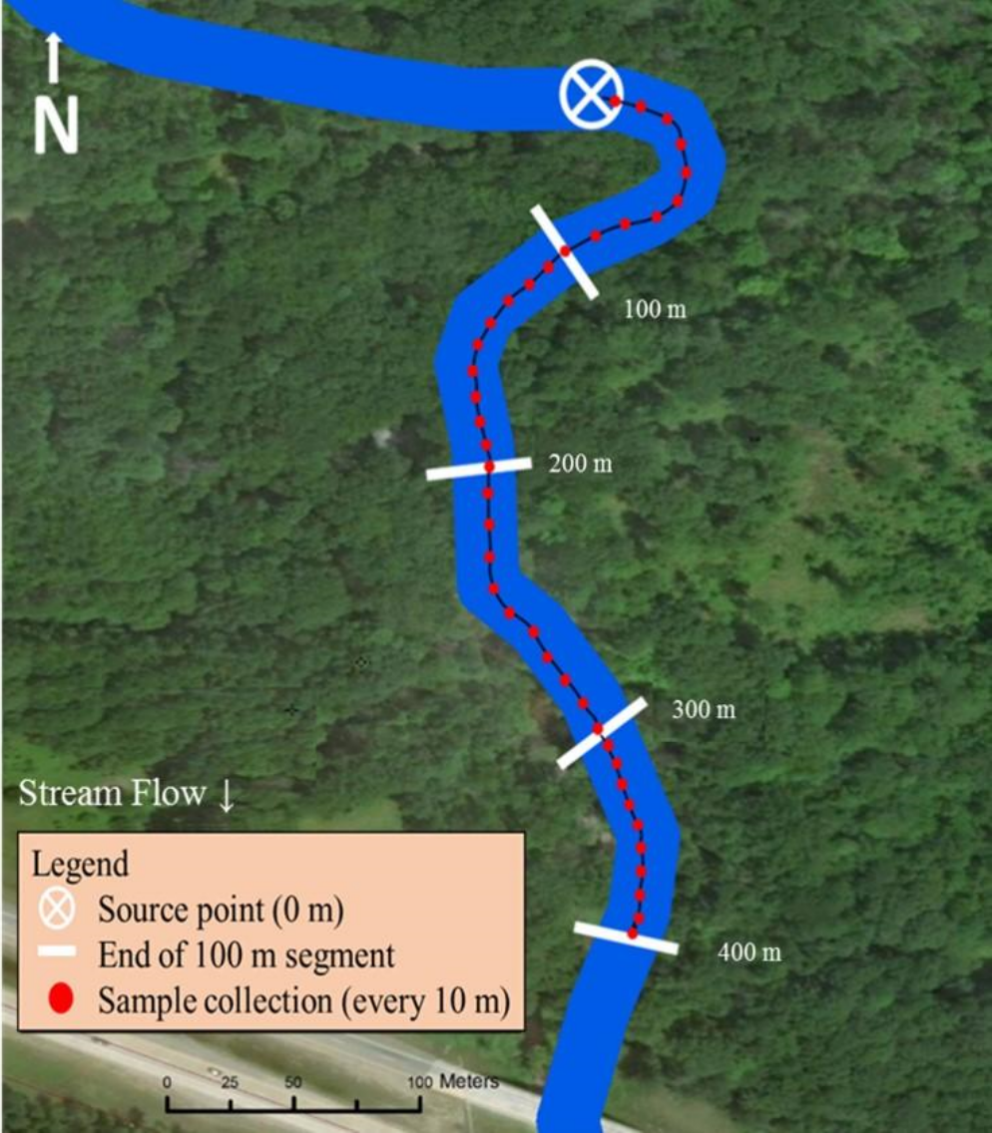
Sampling design used in Bluff and Black Creeks, Michigan, USA, to detect swine DNA in free-flowing streams. Source point represents the location where swine DNA was introduced to the stream. Data source: Esri DigitalGlobe.

The midpoint of both stream sampling transects was strategically selected for road or trail accessibility by truck or ATV. The sampling scheme was then based on equal distribution of trail cameras up and downstream from the pre-determined midpoint (200 m; Fig. 2). If a road or bridge occurred at the midpoint, the location was moved upstream so the end of the sampling transect (400 m) ended ≥100 m upstream from the man-made structure.

Metal fence posts were placed on opposite streambanks to mark the location for artificial insertion of swine DNA. Additionally, one post was placed every 100 m downstream for a total of 400 m to mark the start location of each 100 m segment. Flagging was used along the stream bank to mark 10 m increments within each segment and these locations were GPS recorded (Garmin GPS 12 XL; Garmin Ltd., Olathe, Kansas; Fig. 2).

There is a long history of interbreeding among domestic and wild pigs (McCann et al., 2018), therefore, feral and domestic swine DNA could not be differentiated on the molecular level at the time of this study. Therefore, DNA specimens (legs and/or hides) were acquired from feral or domestic swine culled by the United States Department of Agriculture—Wildlife Services in Michigan, or from a swine processing facility. Each specimen was frozen and subsequently thawed for 24 h before insertion into a stream. To simulate a swine crossing a stream, specimens were attached to a rope pulley system between the fence posts and pulled through the stream four times at the source point, and then the specimen was bagged, removed from the site, and disposed.

Water samples were collected with at least 18 days between each DNA insertion, ensuring DNA degradation in the environment between sampling events (Dejean et al., 2011; Thomsen et al., 2012; Piaggio et al., 2014). Samples were collected from the water surface in the middle of the stream using a telescopic dipper (1.5–3.5 m Conbar Telescopic Dipper; Forestry Suppliers Inc., Jackson, Mississippi). The ladle was dipped in an upstream motion while collecting the minimum amount of water needed (∼50 ml), and extra stream water was placed in an 18.9 L bucket for disposal. To avoid cross contamination of samples between subsequent sampling of each 100 m segment, the telescopic dipper was sterilized with a 20% bleach solution (Goldberg et al., 2013; Jane et al., 2015; Williams, Huyvaert & Piaggio, 2016) for 2–5 min then rinsed multiple times with distilled water into the 18.9 L bucket.

Sterivex filters (Sterivex—GP capsule filter pore size 0.22 µm; Millipore Sigma, Darmstadt, Germany) were used for collecting and transporting DNA based on prior eDNA extraction efficiency studies (Spens et al., 2017). Because filters can clog when sampling turbid waters, 2 Sterivex filters were used for collection (250 ml per filter; Piaggio et al., 2014). To ensure that swine DNA was not naturally occurring, prior to swine DNA being manually introduced into streams 500 ml of water was strained through filters at the source point (designated as *T* = 0). To determine whether DNA was detectable at the source point immediately after DNA insertion, another 500 ml of water was strained through filters (designated as *T* = 1). This process was repeated two hours later (*T* = 2) to assess if DNA persisted at the source point. After *T* = 2 sampling, collection continued downstream for a total of 400 m (Fig. 2). Every 10 m within a 100 m segment, one 50 ml sample was strained through a filter for a total of 500 ml collected from each 100 m segment. Sterivex filter water samples were filled with Longmire’s lysis buffer using a 3 ml syringe (Renshaw et al., 2015) to preserve DNA captured in the filter, capped, labeled individually, and then wrapped with parafilm. Filters were stored at room temperature until DNA extractions could be completed. After each sampling event, all samples were directly transported to the genetics laboratory for molecular processing.

### Stream measurements

Water temperature, velocity, pH, and turbidity were measured during each DNA sampling event. Water temperature, pH, and turbidity were collected from shore using the telescopic pole and dipper during collection of water samples. Temperature and pH meters (Oakton pH 5+ and 6+ Meters, pH/Temperature/mV; Oakton Instruments, Vernon Hills, Illinois) were calibrated prior to sampling each day and data were recorded at the source point and at every 100 m segment downstream (Fig. 2). Turbidity, measured in nephelometric turbidity units (NTU), was monitored using a turbidity meter (Oakton Turbidity Meter; Oakton Instruments, Vernon Hills, Illinois) by collecting a small water sample (>10 ml) in a small glass vial and placing it into the meter. The turbidity meter was calibrated before each sampling event. Turbidity was collected from the middle of the stream at the source point and every 100 m downstream (Fig. 2). The stream bottom or water flow could not be disturbed by researchers during DNA sampling so velocity was collected at the end of each sampling event. Velocity was measured using a flow probe and average velocity function (Global FP111 Flow Probe; Geo Scientific Ltd., Vancouver, British Columbia, Canada). Average velocity was determined by moving the flow probe smoothly and evenly back and forth from top to bottom of the water column for 20–40 s. Sampling the 400 m transect took an average of 4 h 45 min and changes may have occurred in flow from the beginning of a sampling event to the end.

### Laboratory sample processing

DNA extraction and digital droplet polymerase chain reaction (ddPCR) (BioRad QX200 Droplet Digital PCR System; BioRad Laboratories Inc., Hercules, California) were used to process field samples in the molecular lab. Total DNA extraction from Sterivex filters followed the protocol developed by Spens et al. (2017). Extracting DNA from the Sterivex filters necessitated approximately 48 h during which DNA was extracted from the Longmire’s lysis buffer and the enclosed filter using a Qiagen DNEasy kit (Qiagen, Inc., Hilden, Germany). During the second day of the extraction protocol, the DNA found in both the Longmire’s lysis buffer and enclosed filter were combined and held at −20 °C until they were analyzed by ddPCR. Sterivex filters underwent DNA extraction individually and then samples from the 2 filters used for collection were pooled for each individual sample event (i.e., *T* = 0, *T* = 1, *T* = 2, and each 100 m segment). A subsample of the pooled DNA per segment was used for ddPCR and the remaining DNA was archived at −80 °C.

The ddPCR platform partitions each sample into ∼10,000–20,000 individual replicates (droplet) for each DNA extraction analyzed. The platform targets and results in an actual count of target molecules (Nathan et al., 2014). Target *Sus scrofa* primers and a species specific hydrolysis probe for ddPCR were previously developed by Williams, Huyvaert & Piaggio (2017). Targeted swine DNA presence was measured by the amount of target DNA copies per microliter (copies/µl). Every ddPCR plate (i.e., each time a group of samples were screened/analyzed) included three positive swine controls from genomic DNA (tissue extraction DNA) and three negative controls (blanks; ddH2O). In all analyses, the positive control tested positive and the negative controls screened negative, demonstrating no issue with contamination in the processing of the DNA samples in the laboratory. Water blanks were included for DNA extractions, analyzed as normal samples, and no water blanks tested positive for swine DNA. For this study, any sample with a minimum of one positive droplet per µl indicated a positive sample for the presence of swine eDNA. Positive samples were replicated in order to demonstrate repeatability of the data. However, simply based on cost per sample and timing for this research, we did not repeat processing of all negative study samples.

### Data analyses

Multicollinearity of predictor variables was assessed by computing a Variance Inflation Factor (VIF; Cohen et al., 2003) for each predictor variable from a fully parameterized general linear model (binomial family). The full model included swine DNA detection (binary response, yes or no) as the response variable and stream type (as a factor), distance downstream, stream velocity, water temperature, water pH, discharge, and turbidity as predictors. Predictors with VIF >5 were sequentially removed from the model, with highest VIF removed first. Distances downstream from the swine DNA source point included 0 m (*T* = 1), 100 m, 200 m, 300 m, and 400 m. Stream was included as a factor because Bluff and Black Creeks represented variable habitat types and stream characteristics. Parameters from the final generalized linear model were significant if 95% confidence intervals (CI) did not overlap 0. All analyses were completed using packages lme4, regclass, and ggplot2 in R Version 3.6.1 (R Core Team, 2019).

## Results

All samples screening positive for presence of swine DNA were re-tested to validate the previous positive reactions and at no time did the repeated reactions fail to detect swine DNA. Additionally, all controls, completed for each plate of ddPCR run, included three positive reactions and three negative controls. All positive controls were found to be positive, and all negative controls were found to be negative throughout the entire process.

No physical sign or photos of feral swine were recorded around field sampling sites during the study. Swine DNA was introduced 14 times between August 2017 and April 2018; 980 total water samples were collected from Bluff and Black Creeks. One downstream sampling event for Black Creek in September 2017 did not occur because the stream lacked flowing water, but data were collected for *T* = 0, *T* = 1, and *T* = 2. One sampling event was excluded from analyses due to contamination of the control sample (*T* = 0) therefore 13 sampling events informed regression modeling.

**Figure 3:**
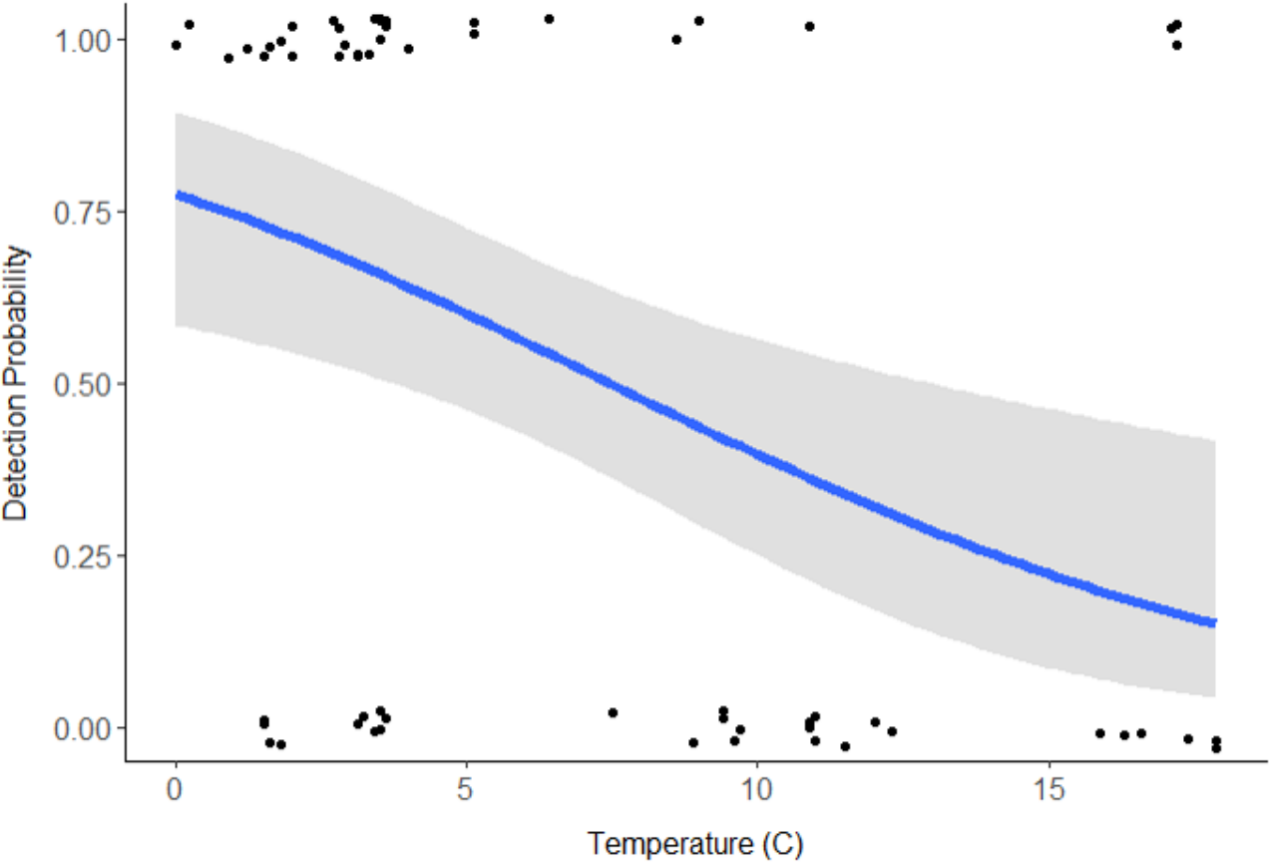
Detection probability of swine DNA by water temperature (°C) for Bluff and Black Creeks, Michigan from generalized linear model. Shading represents 95% confidence intervals.

Discharge (VIF = 15.09) and pH (VIF = 9.58) were deemed redundant, hence the final model included stream type, distance downstream, water temperature, velocity, and turbidity (Table 1). Distance downstream and velocity were hypothesized to influence movement of swine DNA, so they were examined as an interaction; however this was not supported by the model (*β* =  − 0.59, 95% CI [−2.139 to 0.883]). Water temperature was the only variable with a significant effect on swine DNA detection (*β* =  − 0.205, 95% CI [−0.346 to −0.085]; Table 1). Detection probability for swine DNA decreased as water temperatures increased (Fig. 3). Estimated detection of swine DNA in flowing streams in Michigan ranged from 0.05 to 0.08 depending on water temperature (Fig. 3). Detection probability declined to <0.50 at temperatures >7.5 °C (Fig. 3).

**Table 1:** Generalized linear model coefficient estimates, standard errors, and upper and lower 95% confidence intervals (CI) for detection of swine DNA in Michigan streams.

	Coefficient estimate	Standard error	95% CI lower limit	95% CI upper limit
Stream type	−0.323	0.660	−1.639	0.983
Distance downstream	0.323	0.216	−0.087	0.770
Water temperature	−0.205	0.066	−0.346	−0.085
Velocity	−0.178	1.842	−3.688	3.648
Turbidity	−0.122	0.113	−0.368	0.093

## Discussion

Use of eDNA techniques to detect aquatic species is common (Jerde et al., 2013; Deiner & Altermatt, 2014; Takahara, Minamoto & Doi, 2015; Turner, Uy & Everhart, 2015; Stoeckle, Soboleva & Charlop-Powers, 2017); however, few studies have applied this technique to detect terrestrial mammals. Coyote (*Canis latrans*) eDNA was successfully detected in drinking water sources at a captive facility and the authors suggested this may have broader application for detection of free-ranging terrestrial species (Rodgers & Mock, 2015). Feral swine frequent wetland environments (Mayer, 2009; Gray, 2019) and Belden & Pelton (1975) determined that 43% of wild pig wallows were located in slow moving streams in eastern Tennessee. Swine eDNA was also successfully detected for approximately 14 days in a controlled wallow experiment by Williams et al. (2018). Past research of feral swine movements in Michigan acquired 51,984 GPS locations from eight collared individuals over three years. Marked feral swine were located within 400 m of creeks, streams, or rivers approximately 36% of the time. During the study, feral swine crossed a creek, stream, or river, at least 888 times, or roughly every 1.2 days; including 191 crossings during the winter months (December 21–March 20; S Gray, pers. comm., 2019). Collectively, these studies indicate that feral swine may be an ideal terrestrial mammal for detecting eDNA in free-flowing stream environments.

Swine DNA was detected in free-flowing aquatic systems in a controlled experiment in Michigan. Detection probability decreased with increasing water temperature. Other studies have documented the effects of water chemistry and temperature on DNA degradation (Barnes et al., 2014; Strickler, Fremier & Goldberg, 2015; Lance et al., 2017). In a controlled experiment, Strickler, Fremier & Goldberg (2015) determined water temperature at 5 °C extended DNA detection for bullfrog tadpoles (*Lithobates catesbeianus*) compared 25 °C and 35 °C. Colder water temperatures may limit degradation of swine DNA and/or colder water conditions may retain swine DNA in a more concentrated location (Fremier et al., 2019). The results indicate sampling for feral swine DNA is recommended at cooler times of the year, particularly when water temperatures are below 7.5 °C. Even under these favorable conditions, detection probability is <1.0 and researchers should be cautious due to the chance of false negatives. The probability of false negatives can be reduced with repeated sampling from the same location.

Due to the limited time a terrestrial mammal is expected to utilize water environments, detecting eDNA creates new obstacles for wildlife and molecular biologists. DNA was not detected in 5 of 13 (38%) sampling events at *T* = 1 (immediately after DNA insertion) but was detected further downstream in all instances (i.e., 5 of 5 negative *T* = 1 events). This suggests that stream flow likely facilitates movement of DNA under natural conditions (Deiner & Altermatt, 2014). Fremier et al. (2019) determined that stream hydrology and geomorphology influenced eDNA retention in stream systems. Additionally, sampling ended at 400 m approximately 4–5 h after swine DNA insertion and detected DNA 9 of 13 (69%) times. Deiner & Altermatt (2014) detected DNA >9 km downstream from the source for two species of aquatic invertebrates. It remains untested how far downstream swine DNA would be detectable but it likely differs from aquatic species that continually shed DNA into streams. Future studies should evaluate the distance eDNA can be detected under variable stream conditions and alternative collection strategies, such as collecting samples from deeper within the stream water column, along stream banks, or from sediment (Turner, Uy & Everhart, 2015; Fremier et al., 2019).

Feral swine cannot presently be distinguished from domestic pigs using molecular techniques, so managers attempting to apply the results of this research in areas with documented domestic swine operations should be aware of this confounding factor. However, research on the population structure of feral swine in the United States (Caudell et al., 2013; McCann et al., 2018) may provide future opportunities for employing eDNA techniques to detect and distinguish localized populations of wild pigs for targeted removal.

## Conclusions

Feral swine are among the top one-hundred worst alien invasive species in the world (Lowe et al., 2000), and significant challenges exist in detecting low density populations of feral swine in northern latitudes of North America (Etter, Nichols & Hollis-Etter, in press). Remote sampling using eDNA is one potential tool for detecting feral swine in these environments. This research evaluated the detection probability of swine DNA when introduced into free-flowing streams under natural conditions and successfully detected DNA up to 400 m downstream after introduction. The influence of water temperature, velocity, and pH were evaluated and the research conclusively indicated that swine DNA detection increased with decreasing water temperature. The results show promise for using eDNA techniques to detect a terrestrial invasive species in free-flowing streams, but further research is needed to determine how far detectible DNA travels and how long it persists in stream environments.

##  Supplemental Information

10.7717/peerj.8287/supp-1 Supplemental Information 1 R code

## Additional Information and Declarations

### Field Study Permissions

The following information was supplied relating to field study approvals (i.e., approving body and any reference numbers): The Michigan Department of Natural Resources issued a state land use permit for access (73-2017-013).

### Data Availability

The following information was supplied regarding data availability: The raw data is available at ICPSR: Hauger, Amberly, and Hollis-Etter, Karmen. Environmental DNA in Streams to Detect Feral Swine MI. Ann Arbor, MI: Inter-university Consortium for Political and Social Research [distributor], 2019-09-13. DOI: 10.3886/E109748V3. The code is available as Supplemental File.
